# Can developmental cognitive neuroscience inform intervention for social, emotional and behavioural difficulties (SEBD)?

**DOI:** 10.1080/13632752.2012.757097

**Published:** 2013-01-17

**Authors:** Norah Frederickson, Alice P. Jones, Laura Warren, Tara Deakes, Geoff Allen

**Affiliations:** a Department of Clinical, Educational and Health Psychology, UCL, London, UK; b Department of Psychology, Goldsmiths, University of London, London, UK; c Educational Psychologist; d Head of Specialist Provision; e Headteacher

**Keywords:** neuroscience, callous-unemotional traits, SEBD, intervention, evaluation

## Abstract

An initial evaluation of the utility of designing an intervention to address neuroscience-based subtyping of children who have conduct problems was undertaken in this pilot study. Drawing on the literature on callous–unemotional traits, a novel intervention programme, ‘Let's Get Smart’, was implemented in a school for children with social emotional and behavioural difficulties. A mixed-methods design was used to investigate the perspectives of staff participant-observers in the change process, alongside standardised scores on measures of pupil performance and behaviour. Both qualitative and quantitative results showed reductions in externalising behaviour and improvements in measures of hypothesised underlying cognitive and affective processes. While externalising behaviour improved across subtypes, associated changes in underlying processes differed by subtype, supporting the potential value of neuroscience-informed contributions to intervention planning.

## Introduction

There is great interest among teachers in the potential of neuroscience to enhance educational practice (Goswami 2006; Pickering and Howard-Jones 2007), but considerable debate about the extent to which this potential can be realised (Varma et al. 2008). Obstacles identified by Varma et al. (2008) range from the conceptual to the pragmatic. Conceptual concerns include conflicting philosophies of the disciplines of education and neuroscience and inappropriate reductionism, or at least insufficient consideration of important educational issues such as context. Pragmatic concerns include cost, threats to disciplinary independence and prematurity. The last of these concerns, that application to education may be premature given the present level of knowledge about the brain, has not prevented the burgeoning of ‘neuromyths’ in education (see Geake 2008). Some of these incorrect beliefs about the brain are widely held by teachers (Pickering and Howard-Jones 2007). For example, despite its popularity, there is no neural basis for identifying children by preferred learning style – visual, auditory or kinaesthetic. Neither is there evidence that teaching to preferred learning styles as such is beneficial (Krätzig and Arbuthnott 2006), although there may be incidental beneficial features such as the production of more varied learning materials, with broad appeal and likelihood of engaging a range of students.

To some extent, the prevalence of neuromyths may reflect a currently unmet desire for information from neuroscience research that has clear implications for educational practice. However, such implications are never likely to be derived directly. While brain images have a compelling quality (McCabe and Castel 2008), localising aspects of cognition to particular brain structures cannot, of itself, inform classroom practice. Where the data are correlational, as is often the case, it is not possible even to draw conclusions about causality. Consider the example of dyslexia, an additional difficulty of many pupils with social and emotional behavioural difficulties (SEBD) (Carroll et al. 2005). As Goswami and Szucs (2011) point out, the consistent finding of underactivity in the left parietal–temporal cortex during reading tasks in children with dyslexia cannot be interpreted as demonstrating that underactivity in this brain area causes dyslexia. However, the involvement of the left parietal–temporal cortex in phonological processing does support the behavioural test data implicating this cognitive ability as a crucial area of difficulty in dyslexia (Turkeltaub et al. 2003). Furthermore, the finding that intensive phonologically based interventions can be successful for some children with severe dyslexia in both improving reading performance and ‘normalising’ brain activity (Simos et al. 2007) establishes the neuroscientific data as at least a valuable complementary source of evidence in evaluating programme efficacy, as well as identifying intervention targets.

In as well-established an area of research and practice as dyslexia, it is perhaps not surprising that the identified intervention target, phonological processing, had already been much investigated by researchers in developmental cognitive psychology. Neuroscience research in a more recently investigated area, specific difficulty with numeracy or dyscalculia, has identified a core deficit in an area not previously targeted by educational interventions: numerosity: the inherited ‘number sense’ that enables estimation ‘at a glance’ of sets of up to four numbers. As a result of this research, interventions have been developed aimed at strengthening number sense, rather than more traditional foci of remedial maths programmes, for example, on teaching number bonds. Promising initial results have been obtained both from individualised special needs teaching and educational software programs using this approach (Butterworth, Varma, and Laurillard 2011). It is clear from published accounts of this work that even if neuroscience can contribute by identifying a novel intervention target, the involvement of educationalists is crucial in developing programmes based on the neuroscience, which utilise sound educational principles (for example, relating to pupil engagement), and in evaluating the achievement of important educational outcomes. In this article we report work in a less well-developed area still, describing the development, piloting and initial evaluation of a neuroscience-informed programme aimed at improving the social and learning behaviour of pupils in a primary school for SEBD. First we briefly review the neuroscientific research which most centrally informed programme development.

### Neurocognitive profiles in SEBD: the case of callous–unemotional traits

In recent years, neuroscience research with children and young people has identified a number of pathways that may lead to problematic social behaviour (Viding and Jones 2008). Functional magnetic resonance imaging (fMRI) studies that have compared the responses of groups of children with and without conduct problems when viewing mildly threatening emotional scenes have identified two different response patterns. On the one hand, generic groups of children with conduct problems have been found to show reduced anterior cingulate activation (suggesting poor emotional regulation) and increased amygdala activation, partly related to accompanying anxiety (suggesting heightened emotional reactivity) (see Sterzer and Stadler 2009 for a review). Many such children are found to have experienced environmental adversity and their behaviour is often characterised by reactive aggression.

By contrast, a subgroup of children with conduct problems have been identified who more often engage in proactive aggression, showing reduced emotional responsiveness to others, including diminished affective empathy and remorse. Such callous–unemotional (CU) characteristics have been found to have a stronger genetic basis and be more prevalent in those children whose problems are of early onset and high severity (Viding et al. 2005). fMRI studies of children with high CU levels indicate lower amygdala activity in response to others’ distress (Jones et al. 2009). In addition, high CU levels are associated with atypical ventromedial prefrontal cortex responses to punishment (such as losing points), and with disrupted integration of amygdala, orbitofrontal cortex and caudate function during reinforcement learning (Finger et al. 2008, 2011). These findings suggest a functional neural basis for the reduced effectiveness of sanctions (but not reward) reported for this group. They may also help to explain the balance of evidence that suggests poorer response by children who have high CU levels to traditional interventions, such as behaviour therapy (Haas et al. 2011), or parent training (Hawes and Dadds 2005), where relative responsiveness to sanctions in the form of time out was particularly reduced.

Viding et al. (2011) have discussed a range of implications of these neuroscience findings for education. For example, it may be more effective to intervene with children who have SEBD and high CU levels in ways that appeal to their self-interest and focus on reward, rather than employing sanctions-oriented strategies or attempting to induce empathy for victims. For children with SEBD who do not exhibit CU traits, a focus on emotional regulation and behaviour management may be more appropriate. Given that children with different profiles will usually be learning side by side in the same classroom contexts, the challenge is to develop programmes that can be used to address a range of needs effectively.

### Programme development

*Let's Get Smart* (LGS), a flexible, multi-stranded behaviour intervention programme, was developed by the third author, an educational psychologist, in response to increasing requests for support for children with early-onset and more challenging aggressive and antisocial behaviour. Casework experience highlighted apparent differences in the emotional responses, social interests and behaviour of those children who engaged in the most damaging antisocial behaviour, e.g. persistent bullying.

The emerging neuroscience research evidence reviewed above, which was accessed during the third author's professional doctorate studies, indicated the potential value of systematically planning for children with different neurocognitive profiles, in particular those with high CU levels. LGS took as its starting point the range of individual differences in children's cognitive and socio-emotional profiles, and identified strategies that could address both antisocial behaviour which is planned and goal-directed, as well as that which is less controlled and more highly influenced by contextual factors, e.g. the actions of others. A key aim of LGS is to increase a child's motivation to attend to, and have regard for, other people's perceptions, needs and preferences. This is regarded as fundamental, as it is the mechanism through which children come to internalise the values and rules of society, but is thought to be compromised in particular where children have high CU levels (Dadds et al. 2011).

LGS also embraces the neuroscience research which highlights the importance of executive functioning (EF) and emotional regulation for managing behaviour and learning (McCloskey, Perkins, and Van Divner 2009; Viding and Jones 2008). Consequently, strategies are used which aim to increase children's capacity for inhibition, attention, working memory, flexibility and self-monitoring: the overarching goal being for the pupils to function independently in contexts that are increasingly demanding emotionally, socially and cognitively. In recognition of the fact that many children with behaviour problems have weaknesses in their EF and language skills (Law and Plunkett 2009), a range of visually supported intervention tools are incorporated. A summary of programme target areas, the underpinning research rationale for each and the programme components used to address each target area is provided in Appendix A, together with further details of a number of exemplar LGS strategies. While some strategies have been adapted from existing behaviour modification tools (e.g. emotion thermometers and quiet time), the design of others has been specifically informed by research on the strengths and needs of children with high CU levels (e.g. the LGS approach to communication, the use of thought–behaviour chains and providing immediate rewards with removal of sanctions). The aim was to be able to address the full range of key individual differences in pupils’ social–emotional profiles.

Behaviour for learning targets, which are regularly updated, are recorded on an LGS action plan, along with strategies and other significant information, e.g. which LGS tools the child will be encouraged to use. The plan is shared with other key adults, so that coaching and support can be provided across the school day. An important component of the programme involves training adults to use the LGS approach to communication, which contains language use (e.g. ‘Smart Body’ – see Appendix A, section on thought–behaviour changes) designed to promote clarity and reduce the incidence of unwanted behaviour. The training also enables schools to consider ways in which they might modify curriculum delivery, the structure of the school day and their behaviour polices. Children are actively involved in monitoring their progress towards their targets, to improve their self-awareness and capacity for reflection. Immediate, clear, detailed feedback is given to enhance progress and a carefully structured reward system is included to maximise motivation.

### School context

The pilot school is a special school for children experiencing SEBD to the extent where they have been judged unable to learn within a mainstream environment. At the time the project started, the school had been open for 12 years and had a successful record of managing and changing children's learning and behaviour, reintegrating approximately 20% of pupils to mainstream schools. However, school staff recognised that the profile of needs of the pupils entering the school had changed to the extent that progress in learning and behaviour had become more difficult to maintain. For an increasing number of pupils, the strategies that formed the core of good practice in the school, based within a framework of social learning (Green and Ablon 2006; Kazdin 2003) and attachment theory (Boxall 2002; Kennedy and Kennedy 2004) had become ineffective. Incidences of extreme behaviour, leading to injury and damage, had become more common and widespread across a greater number of pupils across the age ranges. Four pupils in particular presented such extreme behaviours at the ages of 6 and 7 that they were at risk of permanent exclusion.

The *Let's Get Smart* programme was introduced to the school when the third author became the link educational psychologist. An initial pre-pilot trial with a small group of pupils presenting with extreme behaviour produced positive findings, on the basis of which the local authority decided to proceed with an externally evaluated, school-wide trial. At the start of the pilot training for all school staff was provided by the third author, to begin to develop a theoretical understanding of the different pupil needs profiles and to familiarise staff with the rationale underpinning the LGS principles, strategies and tools. A Specialist Provision was created to work, initially, with the four most challenging pupils, who were identified as requiring access to more intensive LGS work. This included daily participation in LGS curriculum work, to develop their social–emotional functioning and capacity for inhibition, attention, working memory, flexibility and self-monitoring.

Staff coaching and consultation continued for the duration of the pilot to ensure the consistent application of the LGS strategies and to develop with school staff modifications and additional strategies based on the programme principles to address specific issues as they arose. For example, during the pilot, observations of the impact of environmental factors on a child's capacity to regulate their emotions and behaviour led to the structure of lessons being changed, by subdividing them into 15-minute segments and introducing short breaks between learning activities. Once the Special Provision staff had secured their understanding of the programme principles and felt confident in their use of its tools, they themselves began to provide coaching and consultation to their fellow teaching and support staff in the main part of the school.

The school's approach to behaviour management was changed, and staff implementation of the LGS approach to communication began by use of a bank of key phrases (e.g. ‘Smart Body’, ‘Ready to Learn’, Quiet time’ – see Appendix A) taught during the initial training and aimed at increasing the children's motivation to modify their behaviour to take account of adult feedback, by including reference to the children's self-interests. Later on, staff were able to apply LGS principles in order to modify and add to their bank of phrases. The children also contributed in shaping project implementation. For example, during work on inhibition training one of the children commented on his difficulties in stopping himself thinking, doing or saying certain things, saying that it was like having a ‘stop button’ in his head that did not work properly. The child's analogy was considered very helpful, and the phrase was widely adopted throughout the project when referring to inhibition activities.

## The pilot evaluation

The evaluation of the pilot reported in this article was funded by the local authority in order to ascertain the extent of positive change for staff and pupils achieved in the first year of implementation. A mixed-methods design (Creswell and Plano Clark 2007) was adopted in order to capitalise on the role of staff members as participant observers in the change process, while also utilising standardised measures of pupil performance in collecting information on change over time to address accountability requirements. The qualitative component of the study involved semi-structured individual interviews conducted with school staff by a university researcher in the summer term at the end of the first year of the pilot. The quantitative component involved completion by staff of standardised measures of child behaviour and performance on two occasions: in the autumn term prior to the introduction of the programme to the school, and again in the summer term in order to evaluate change in standard scores.

## Qualitative investigation of staff perspectives

The purpose of this component of the study was to explore staff perspectives on the changes they had experienced in their work, and observed more generally in the pupils, following the implementation of the programme. In addition to this descriptive information, evaluative information was sought on what had, and had not, worked well.

### Method

#### Data collection

Semi-structured interviews were held with 13 members of staff (5 teachers, 6 teaching assistants, deputy head and head teacher). Although not all staff were interviewed, staff across different areas of the school were included. Interviewees were assured of confidentiality and of anonymity in reporting. Semi-structured interviews allowed focused, open communication where not all questions were necessarily planned ahead of time, so providing opportunities to follow lines of enquiry that appeared particularly informative or important (Wengraf 2001). The interviews were based around the questions about changes resulting from the pilot.

- What has changed for you since September?
- What has changed for the pupils?
- What has worked well?
- What has not been so effective and could be improved?

#### Data analysis

The interviews were audio recorded and transcribed. Content analysis of the transcripts was based on procedures described by Vaughn, Schumm, and Sinagub (1996) as follows.

(1) Identification of key themes or ‘big ideas’ within the data, following reading and re-reading of each transcript.
(2) Identification and highlighting of units of information (phrases and/or sentences) relevant to the research purposes.
(3) Selection of category headings to sort and group these units of information.
(4) Coding of units of information according to category headings, to enable most of the units to be placed within a category. A software package for qualitative data analysis, Atlas.ti (Muhr 2004), was employed.
(5) Negotiation between the researchers to agree the category headings which most economically accommodate the relevant units of information.
(6) Review, revision and cross-validation of the categories generated in the first phase of data analysis through participant verification.

### Results

Three overarching themes were identified in the data: changes for staff, changes for pupils and broader issues affecting programme implementation. The themes and subthemes identified in the first two of these areas are presented in detail in this section, and illustrated with direct quotations from the interview transcripts. A number of broader issues affecting programme implementation are then summarised and information from documentary sources provided in the process of transcript verification outlined.

#### Changes for staff

The themes and subthemes identified from the transcripts about changes for staff resulting from the programme are shown in Table 1. There were three main themes: the identification of a new focus on pupil needs, the evaluation of changes in strategies used by staff with the children and the evaluation of broader practice changes, including teamworking.

**Table 1.** Changes for staff theme: number and percentage of sub-theme occurrences.

Sub-themes	Number	Percentage
**Focus on pupil needs**		
Inhibition	6	4.9
Working memory	3	2.4
**Strategies**		
Communication	12	9.8
Emotion thermometers – positives	10	8.1
Emotion thermometers – difficulties	2	1.6
Quiet time – positives	6	4.9
Quiet time – difficulties	3	2.4
Remove sanctions – positives	3	2.4
Remove sanctions – difficulties	2	1.6
Smart bodies	4	3.3
Stop button	11	8.9
Targets – positives	21	17.1
Targets – difficulties	3	2.4
Thought chains	2	1.6
**Broader changes for staff**		
Changing practices – positive	8	6.5
Changing practices – difficulties	3	2.4
School/team working – positive	13	10.6
Whole school working – difficulties	11	8.9
**Total**	**123**	**100%**

Staff identified a new focus on pupil needs relating to the project assessment data, in areas such as inhibition and working memory: ‘It's a really interesting thing because working in this way, going back to working memory and inhibition and that sort of thing you get to find the gaps that a child might have that you might not necessarily see in the way that we had been working.’

Many comments were made about new behavioural strategies that respondents had found successful with pupils, for example:

Communication: ‘I think the language is very important, the way in which you speak to the children and relating it back to their target work’.

Emotion thermometers: ‘The kids themselves are much better at saying “I'm a 3, I need some quiet time” and just taking a couple of minutes and being back on task within 5 minutes and it being a successful lesson.’

Stop buttons: ‘We use stop button a lot and quiet time. We had quite a few sessions on that to start with and now we just say “stop button” and they seem to know what you are talking about. Rather than saying “stop moving your chair or table” we can just say “stop button” and they know.’

Quiet time: ‘I think for me it is quite easy to explain to them that “I need you to take quiet time”. Because it seems more positive – I am trying to help you, why don't you take some time, it's not a punishment, you do need to walk away.’

Removal of sanctions: ‘… and I think the children have really picked up on the fact that we don't just sanction them and tell them they are naughty any more and they respond to that.’

Thought chains: ‘So things like using the thought chains so that they understand what we are trying to do with them as well as us being really clear about what we are trying to change and how we are trying to get there with them. That has been the thing that has made the biggest difference.’

There were several comments about targets being more effective than effort grades, which had been used previously, for example: ‘I think it makes it easier for the children to understand because if you ask them about their targets they all know what their behaviour target is … because we used to do effort grades and there used to be arguments about whether it was a 3 or a 4, so it's easier in that respect.’

There were some reservations about the strategies, although these were relatively few, for example:

Emotion thermometers: ‘The temperature thing, we do use it, but probably not as regularly as we should. Partly for me on a personal level is that I don't know where to put it [incorporate it in a lesson].’

Removal of sanctions: ‘But I think there are times when you still need a sanction – in society there will be sanctions, if someone does something wrong – if you commit a crime you will get a fine or be sent to prison – and if you cut out all sanctions they are never going to know when they get out there in the big wide world, they won't understand that.’

Difficulties in implementing targets: ‘But if you have two children doing the same thing inappropriately, if that's one child's target not to do it, and not the other child's then obviously it's a bit difficult. Because they might say “well he was doing that as well” and that's not their target!’

Many comments were made about the broader positive changes for staff, in particular the benefits of team work: ‘it's good team work; you could be banging your head against a brick wall for two weeks thinking why isn't this behaviour strategy working and then you can go to someone else and have a conversation and you can say we need to think of something else.’

Teaching assistants reported increased involvement in team decision-making: ‘you have a lot more kind of say in how things go, for example what we are going to do in the lesson, how we are going to do the lesson and a lot of the planning as well, it's been very interesting to have input into that as well’ (TA).

Some staff suggested they had not been entirely sure the new practices would work when the project started, but had changed their opinion having seen the effects:

… the ignoring and things like that I found quite difficult to begin with because I'm not like that, it's not in my nature – but I can see it works.

I am absolutely in support of all this now, but I wasn't convinced at the start

However, there were some reservations about the full integration of the programme into the whole of the school, and about difficulties staff had experienced in accommodating new practices:

Although we get together at staff meetings we don't know an awful lot of what goes on in [the Special Unit] and it is somewhat like they are putting everything into this new project and it feels like some of the school are only doing bits and bobs and we are not getting any overlap.

I think one of the difficulties – we have a migrant population, we have a lot of children in during the course of the year – the difficulty is when you have children who know how to do it a little bit and a new one in where all the behaviours come out and you have to go through it all again with the others.

#### Changes for pupils

The themes identified from the transcripts about changes staff perceived pupils to have made as a result of the programme are shown in Table 2. These comments were mainly positive, although there were some comments about pupils experiencing difficulties in coping with the changes – this was mainly in relation to pupils in Year 6.

**Table 2.** Staff perceived changes for pupils theme: number and percentage of occurrences.

Sub-themes	Number	Percentage
Decrease negative behaviours	8	12.72
Effects for special unit pupils	13	20.67
Effects for main school pupils	7	11.13
Pupil self regulation & management	28	44.52
Video work	4	6.36
Coping with change – difficulties	3	4.77
**Total**	**63**	**100%**

The most common theme was about differences in how children had been enabled to self-regulate and manage their own behaviour:

they have a much better understanding of the way they work and function like they understand which situations they can deal with and which they can't, so it's given them a better understanding of their own behaviours and why they work that way and the ways in which they can control their behaviours.

They are taking more responsibility for their own emotions and for the child to be able to understand their emotions and why they are feeling like that and not let it spiral out of control.

Some staff perceived that there had been a decrease in negative pupil behaviours:

I am not using as much restraint … I feel that I am involved in far less physical involvement with the pupils – I think things are being resolved in class and are being de-escalated more effectively.

They are learning far more of the time than they were, and reducing the overloaded behaviours and the big kick offs …

It's huge that the others can get on with their lesson and when the child comes back [from taking quiet time], they can get on with it, even if they have missed five minutes.

Specific mention was made about the successes of the pupils in the special unit.

X, his life has changed, his whole way of being has changed – he is a different person … I meet him, he is smiling, he is self contained, he is looking as if he may have the core of a locus of self control, as if he may just have the beginning of feeling there's something in me that will stop me from doing this, and that was absent before, it just wasn't there, and that will change his life.

Where video work had been used, it was considered to have been successful:

when we have used it with X it has been so successful because he really has developed a sense of ‘when I behave like this, this is what other people think and see, so I'm not going to get [desired outcome] if I behave like it.

There were some comments about pupil difficulties in coping with the changes which were focused on the Year 6 class:

I think there is a lot of success, but for the children who have been here a long time under the old system I think it's harder to take on.

#### Programme introduction and implementation

The third set of themes identified from the transcripts concerned more general challenges experienced in programme introduction and implementation. A number of these related to issues, such as staffing and building work, which coincided with but were not perceived as changes resulting from the pilot and are not reported here. However, a strong sense was conveyed that the pilot study had involved, and continued to involve ongoing change. Interviewees commented on how working practices have changed and evolved during the year.

… we have constantly had to tweak things and change things and come back together and go ‘this really isn't working’: we've been trying for a while and we put our heads together and think of something else. So throughout the whole time we have been changing, but it's not big changes, just tweaking behaviour management for each individual child.

Comments were made about looking forward to the next academic year and using the strategies from this first year to be able to extend the project and consolidate practice:

It's been a brilliant year, yes. I think the footings that we have put in are amazing. It's been really hard work – and getting over that initial hump to get going and have ideas formed about where we want to go has been really hard and we need to just keep going.

#### Supplementary documentation

In the course of transcript verification by participants a number of documents were provided in elaboration of points discussed in the interviews. These provided information on exclusions and incidents of notifiable damage. In each case there was a substantial decrease in the year following the introduction of the programme, compared to the previous year. The number of fixed-term exclusions fell from 32 to 7, while incidents of damage to buildings/fittings/furniture fell from 19 to 3.

In addition, an Ofsted report relating to a visit in the year following programme implementation was provided. The work reported in this article was mentioned in the following extracts:

Working closely with [X University], the school has gained national prominence for its work in recognising and addressing the weaknesses many pupils have that inhibit their ability to learn in a conventional manner. (p. 4)

Evaluations carried out by [X University] show significant improvements in pupils’ social awareness and their understanding of morality. (p. 6)

The work with [X University] has seen significant improvement in the outcomes for pupils. (p. 8)

## Quantitative investigation of changes in behaviour and performance

The purpose of this component of the study was to investigate specific questions about changes in pupil behaviour and performance over the 9 months following introduction of the intervention.

(1) 
  (a) Would standardised scores on measures of externalising, disruptive and aggressive behaviour show significant improvement?
  (b) Would these behavioural improvements be greater for children with low CU levels, in accordance with previous reports of greater intervention resistance in those with high CU levels?
(2) 
  (a) Would standardised scores on measures of executive functions relating to behavioural regulation and metacognition show significant improvement over the school year?
  (b) Would any differences in executive function be found between children with higher and lower CU levels?
(3) Would changes in performance on different indicators of cognitive and affective functioning be associated with improvements in externalising behaviour for children with higher and lower CU levels?

### Method

#### Participants

At the beginning of the school year, parents were informed by the school by letter about the new programme and opt-out parental consent was obtained for the collection of evaluation measures. As the measures in this study were teacher-completed and did not involve direct pupil participation, opt-out parental consent was considered appropriate. No parent withheld consent so all 35 pupils at the school were initially recruited into the project. Due to alternative placements and house moves, complete information at both time points was available for 29 pupils. Of these 29 pupils, 27 were male and the group had a mean age of 8.72 years (SD = 1.71), with 6 being in Key Stage 1 (5–7 years) and 23 in Key Stage 2 (8–11 years).

#### Measures

The following measures were completed by class teachers on two occasions, once in the autumn term and once in the summer term.

*Inventory of Callous–Unemotional Traits (ICU; Kimonis et al. 2008).* The ICU is a 24-item scale, which assesses three key areas considered to underlie CU traits: ‘Uncaring’ (a lack of caring about performance), ‘Callousness’ (a callous attitude towards others) and ‘Unemotional’ (i.e. lack of emotional empathy and lack of emotional expression). The highest possible total score is 72, with higher scores indicating greater problems. Excellent internal consistency reliability (α = .92) has been reported for the teacher-completed ICU (Vaughn et al. 2011), as has strong validity data (Roose et al. 2010). In order to explore possible difference between children with low and high CU scores, the data collected in the Autumn term were subject to a median split. The descriptive statistics for the sample and each of the CU groups on this measure are shown in Table 3.

**Table 3.** Descriptive statistics for the total sample and the high and low CU subgroups on the BASC-II and the BRIEF.

	Total Sample N = 29				High CU N = 14				Low CU N = 15			
	Autumn		Summer		Autumn		Summer		Autumn		Summer	
	*M*	*SD*	*M*	*SD*	*M*	*SD*	*M*	*SD*	*M*	*SD*	*M*	*SD*
BASC-II												
Externalising	75.41	12.44	66.14	15.29	83.86	9.99	74.86	13.91	67.53	8.88	58.00	11.87
Internalising	73.97	17.53	68.38	17.56	74.42	18.16	69.64	19.63	75.53	17.55	67.20	16.00
School Problems	62.03	9.96	57.45	9.75	67.93	8.44	61.64	9.68	56.53	8.07	53.53	8.31
Behavioural Symptoms	77.76	12.46	67.45	15.53	85.21	12.05	75.00	15.07	70.80	8.26	60.40	12.70
Adaptive Behaviour	37.21	6.81	39.48	7.84	31.79	4.84	34.86	5.89	42.27	3.75	43.80	7.01
BRIEF												
Behavioural Regulation	79.86	7.90	74.57	16.06	81.00	9.63	81.71	15.10	78.8	6.00	67.87	14.31
Metacognition	72.10	8.38	68.59	12.28	73.64	7.41	76.21	9.97	70.67	9.21	61.47	9.83
ICU	39.48	13.49	34.86	13.85	51.79	6.15	43.99	10.13	28.00	6.00	26.33	11.27

*The Behaviour Assessment System for Children (BASC-II; Reynolds and Kamphaus 2004).* The BASC-II is a multidimensional assessment system designed to evaluate the behaviour and self-perceptions of children and adolescents aged between 2 and 25. Composite scores include Externalising Problems (comprising Hyperactivity, Aggression and Conduct Problems subscales); Internalising Problems (comprising Anxiety, Depression and Somatisation subscales); School Problems (comprising Attention Problems and Learning Problems subscales), Behavioural Symptoms (comprising Atypicality and Withdrawal) and Adaptive Skills (comprising Adaptability, Social Skills, Leadership, Study Skills and Functional Communication subscales). On all the scales, the average *t*-score range is 41–59. Scores in the ‘Clinically Significant’ range (70 or higher for most scales, but 30 or lower for the last five scales which form the Adaptive Skills Composite) denote a high level of maladaptive behaviour or absence of adaptive behaviour. The BASC manual reports internal consistency reliabilities in the mid .90s for the Behavioural Symptoms and Externalising Problems composites, in the low to mid .90s for the School Problems and Adaptive Skills composites, and the high .80s to low .90s for the Internalising Problems composite. The manual also reports data on the factor structure of the measure and intercorrelations with other relevant measures which offer good support for construct validity

*Behaviour Rating Inventory of Executive Function (BRIEF; Gioia et al. 2000).* The BRIEF assesses pupils’ executive functioning, or self-regulation in their everyday environment. Index scores are Behavioural Regulation which comprises three subscales: Inhibition (defined as ability to control impulses, appropriately stop own behaviour at the proper time), Shifting (defined as ability to move easily from one situation, activity, or aspect of a problem to another as the situation demands, solve problems flexibly) and Emotional Control (defined as ability to modulate emotional responses appropriately) and Metacognition which comprises five subscales: Initiation (defined as the ability to begin a task or activity; independently generate ideas), Working Memory (defined as the ability to hold information in mind for the purpose of completing a task), Planning/Organising (defined as the ability to anticipate future events, set goals, develop appropriate steps to carry out associated tasks or actions), Organisation of Materials (defined as the ability to keep work space, play areas, and materials orderly) and Monitoring (defined as the ability to check work; assess performance during or after finishing a task to ensure attainment of goal; keep track of the effect of own behaviour on others). A total Global Executive Composite (GEC) can be summed from all subscales. On all the subscales, Index scores and GEC, the mean score is 50, and the average *t*-score range is 41–59. *t*-Scores at, or above, 65 on any of the scales or indices indicate clinically significant executive dysfunction. The manual reports good internal consistency reliability on all teacher-rated subscales (α > .8) and convergent validity with other measures of inattention, impulsivity and learning skills.

### Results

Descriptive statistics for all measures for the total sample, and the groups high and low on CU traits, are presented in Table 3. Following preliminary assumption checking which established the suitability of the data for parametric statistical analyses, each of the research questions was investigated in turn.

#### Changes in behaviour

To investigate the first research question, relating to behavioural change, a mixed MANOVA was conducted with the five composite scores of the BASC-II as the dependent variables, CU group (high or low) as the between participants variable, and time (Autumn and Summer) as the within participants variable. Significant multivariate effects were found for time *F*_(5,23)_ = 5.59, *p* = .002, η_p_^2^ = .55, and group, *F*(_5,23)_ = 8.45, *p* = .001, η_p_^2^ = .65, but not for the interaction between time and group, *F*_(5,23)_ = 0.57, *p* = .72, η_p_^2^ = .11. Follow-up ANOVAs were conducted to investigate these significant differences further using Bonferroni-adjusted alpha levels of .01 in the analyses of changes over time and .025 in the analyses of group differences. Significant changes over time were found on the Externalising Problems Composite, *F*_(1,27)_ = 15.11, *p* = .001, η_p_^2^ = .36 and the Behavioural Symptoms Composite, *F*_(1,27)_ = 25.07, *p* < .001, η_p_^2^ = .48. Significant differences between the groups were found on all the composite scores except Internalising Problems. The High CU group scored higher than the Low CU group on Externalising Problems Composite, *F*_(1,27)_ = 23.07, *p* < .001, η_p_^2^ = .46, the School Problems Composite, *F*_(1,27)_ = 13.32, *p* = .001, η_p_^2^ = .33, and the Behavioural Symptoms Composite, *F*_(1,27)_ = 12.86, *p* = .001, η_p_^2^ = .32, but scored lower on the Adaptive Behaviour Composite, *F*_(1,27)_ = 35.29, *p* < .001, η_p_^2^ = .57.

#### Changes in executive functions

To investigate the second research question, relating to change in executive functions, a mixed MANOVA was conducted with the two index scores of the BRIEF as the dependent variables, CU group (high or low) as the between-participants variable, and time (Autumn and Summer) as the within-participants variable. A significant multivariate effect was found for the main effect of group, *F*_(2,26)_ = 5.82, *p* = .008, η_p_^2^ = .31, but not of time, *F*_(2,26)_ = 2.45, *p* = .11, η_p_^2^ = .16, and the main effect of group was qualified by a significant interaction between time and group, *F*_(2,26)_ = 4.27, *p* = .025, η_p_^2^ = .25.

Follow-up ANOVAs revealed significant interactions between time and group for both the index scores: Behavioural Regulation, *F*_(1,27)_ = 6.43, *p* = .017, η_p_^2^ = .19; and Metacognition, *F*_(1,27)_ = 7.19, *p* = .012, η_p_^2^ = .21. Simple effects analyses of these interactions showed that whereas there were no significant differences between the groups in the Autumn on either measure (Behavioural Regulation: *t*_(27)_ = 0.74, *p* = .46; Metacognition: *t*_(27)_ = 0.96, *p* = .35), in the Summer the Low CU group was significantly lower, and therefore performing better, than the High CU group on both measures (Behavioural Regulation: *t*_(27)_ = 2.54, *p* = .017; Metacognition: *t*_(27)_ = 4.01, *p* < .001). While the scores of the Low CU group reduced significantly between the two assessments on Behavioural Regulation, *t*_(14)_ = 2.80, *p* = .013, and Metacognition, *t*_(14)_ = 2.80, *p* < .014, the scores of the High CU group did not show significant change over time on either variable.

#### Association of cognitive and affective change with behavioural change

To investigate the third research question, change scores were calculated by subtracting the scores obtained in autumn from those obtained in summer. Pearson's correlations were first used to examine the association between improvements in externalising behaviour (assessed by the change score for the Externalising Composite of the BASC-II), and improvements both in executive functioning (assessed by the change score for the BRIEF Global Executive Composite) and in CU levels (assessed by the change score for the ICU). Table 4 shows the correlations obtained for the whole sample, from which it can be seen that improvement in externalising behaviour was significantly associated with improvement in both executive function and CU scores. Multiple regression analysis indicated that the combination of the other two change scores significantly predicted improvement in externalising behaviour (*F*_(2,26)_ = 11.52, *p* < .001, Adjusted *R*^2^ = .43), with both variables significantly contributing to the prediction (improvement in executive functioning: *β* = .43, *p* < .01; improvement in CU score *β* = .41, *p* < .05). However, as can be seen from Table 4, when these associations were examined separately for the high and low CU groups, improvement in externalising behaviour was significantly associated only with improvement in CU score for the High CU group, and with improvement in executive functions for the Low CU group.

**Table 4.** Correlations between change scores for externalizing behaviour, executive functions and CU traits.

	Change in Externalising Behaviour score		
	Total Sample N = 29	High CU N = 14	Low CU N = 15
Change in CU trait score	.56**	.62*	.50
Change in Executive Function score	.55**	.44	.82**

*p < .05, **p < 01.

## Discussion

The results of this pilot programme evaluation offer initial support for the further development of the *Let's Get Smart* approach. Significant improvements across the first year of implementation were apparent on a number of measures of pupil behaviour and performance. Changes were mainly apparent in areas particularly targeted by the programme, for example in externalising behaviour, but not internalising behaviour. Changes were also found in measures of the cognitive and affective processes hypothesised to underlie children's externalising behaviour, and the magnitude of these changes was significantly associated with the magnitude of the improvements in externalising behaviour.

Staff perceptions of the changes made by pupils, collected through semi- structured interviews, supported many of the conclusions suggested by the results of the quantitative element of the study. Teachers reported that children were better able to understand and take control of their own behaviour, and that there were fewer instances of behaviour escalation that would previously have required physical restraint or another intervention from a member of staff. Despite a degree of scepticism at the outset, staff evaluation of the changes for themselves, as well as their pupils, was overwhelmingly positive. Furthermore, staff accounts of the changes to classroom and school practices provide evidence of the level of implementation of key programme components.

Programme components were selected to address the needs of children with different neurocognitive profiles. In particular, novel components were incorporated to address the needs of children with high CU scores. For children who had high CU scores at the start of the study, positive changes in CU scores over the course of the year were more strongly associated with behavioural improvements than were positive changes in executive function scores. The converse was the case for children with low CU scores, where positive changes in executive function scores were more strongly associated with behavioural improvement than positive changes in CU scores. These results suggest that this neuroscience-informed way of thinking about children's behavioural needs is relevant to designing appropriately differentiated interventions.

While these broadly positive conclusions may be regarded as encouraging, a number of cautions must be considered. First, the differential associations found for children higher and lower on CU should not interpreted evidence of a linear causal relationship. In the group with low CU scores, the finding that there is a strong association between improvement in externalising behaviour and executive function over the same time period does not imply that the improvement in executive function causes the improvement in externalising behaviour. The converse might be the case, or we may need to consider the role of a third variable not examined in this particular study. One possible mediating variable may be curriculum-based; as externalising behaviour decreases, it may be the case that pupils are more able to participate in curriculum-based activities, and may be developing their executive function skills in this way (McCrae, Mueller, and Parrila 1999).

Second, as only one school was involved, and assessments were only carried out in the year when the programme was implemented, it is not possible to conclude that the improvements occurred because of the programme. It is possible that similar improvements would have occurred during this period in any case, or that any consistent whole-school programme would have produced similar results. In addition, in the absence of follow-up data collection it is not possible to say anything about the maintenance of reported gains. Further research using designs to address these issues is needed. However, the results of the present study provide a prima facie case for recommending that such further research be conducted.

Third, while this evaluation uses mixed methods, there is only one source of systematic information: school staff. A number of the class teachers who completed the standardised measures also participated in the semi-structured interviews. A degree of congruence between the conclusions of the quantitative and qualitative elements of the study is therefore to be expected. While documentary evidence of reductions in exclusions and damage, and from the school Ofsted inspection report, offer some external validation, future research should systematically sample other sources of information.

## Further research

While the content of this intervention programme was developed using a developmental cognitive neuroscience evidence base, and this pilot study offers tentative evidence of reductions in externalising behaviour and CU levels, with increases in executive function performance over the course of one year, it is not possible to relate these behavioural changes to brain structure or activity. Future work should consider pre- and post-measures of brain structure and/or activity in order to examine how far changes measured using teacher reports of observable behaviour are represented in the brain.

Assessment of wider environmental impact would also be desirable, for example through investigating how far reported behavioural changes transfer into the home environment. Many children attending SEBD provision are from disadvantaged backgrounds and have significant difficulties in their home lives (Mattison 2004), and it was beyond the scope of the present study to obtain a comprehensive picture of the home environment, or to gather information from parents/carers, or the young people themselves, at the pre- and post-intervention points. However, future work conducted on a larger scale should seek to collect multi-informant information and to consider the transferability of new skills and behaviour improvement to the home context. It will also be important to investigate whether there are any groups of children for whom this approach may be inappropriate. In this study, the sample of pupils has been too small to investigate differential effects on particular subgroups.

## Conclusions

In summary, this first evaluation study of a developmental cognitive neuroscience-inspired programme of intervention suggests a worthwhile method of bringing about positive behavioural change in a group of young children in SEBD provision. Understanding the neuropsychology and neural underpinnings of CU-type cognitive-affective and behavioural profiles allowed the programme to work to the relative strengths of this group, without being to the detriment of pupils without elevated CU levels. Behavioural improvement occurred alongside improvements in executive functions for some pupils, reductions in fixed-term exclusions from the school and a positive acceptance of the programme by school staff. Initial indications from this pilot are sufficiently promising to warrant the development of a larger-scale evaluation of the programme, and to offer a cautiously positive appraisal of the potential for neuroscience to contribute to educational initiatives for SEBD.

## Appendix 1. Let's Get Smart exemplar strategies

***Adapted emotion thermometer***: This is typically used to help children manage and communicate and their emotions. However, in recognition of the fact that many children with SEBD are unable to identify and understand the impact of emotions on behaviour, the LGS ‘Ready to Learn’ thermometer was developed further so that it:

(a) focuses on behaviours that typically accompany specific emotions, rather than on the emotions per se;
(b) emphasises the relationship between children's thoughts and emotions, and their behaviour, and highlights how this affects their ‘readiness to learn’; and
(c) serves to reinforce and support the child's individual LGS curriculum work, aimed at enhancing their ability to make links between their emotions, thoughts and behaviour, with the goal of increasing their capacity to self-evaluate and self-regulate these.

***Quiet Time***: Within the context of LGS, Quiet Time is used as a supportive strategy to prevent an emotional overload, rather than as a recovery strategy following a ‘meltdown’. It entails the child avoiding, or withdrawing, from a high-stimulation environment for a time-limited period, in order to reduce the risk of a behaviour outburst. Initially, the need for Quiet Time, and its duration, is decided by an adult. However, following LGS training, the decision as to when, and for how long, to take Quiet Time passes from the adult to child. To maximise motivation and progress, the child is rewarded for independent decision-making, e.g. about their readiness to return to learning, and for becoming ready to learn again quickly.

***‘Immediate’ rewards and removal of sanctions***: Prior to the introduction of LGS, rewards were given through ‘golden time’ at the end of the week, and the children had to maintain target behaviour from Monday morning until Friday lunch time.

With the implementation of LGS, the reward system was changed across the school and the sanctions previously in place were removed. One or two specific LGS behaviour targets were identified for each pupil and a reward for achieving them was given at the end of each session. Rewards varied depending on the interests of the pupils; for example, time on the computer, or a game of football. Pupils were taught that if they did something wrong they would not be punished or have anything taken away; however, they would be expected to stop whatever inappropriate behaviour they were displaying, calm down (taking ‘Quiet Time’ as necessary) and go back to the learning activity. Rather than discussing their behaviour at the time, as had been the case before the start of the pilot, it was reviewed with them once they were calm, using LGS principles and strategies, e.g. thought–behaviour chains.

***Thought–Behaviour Chains***: These were used to help pupils develop more accurate, helpful, and socially desirable patterns of thinking and behaviour. Thought–behaviour chains are a teaching tool where, to begin with, the adult elicits the child's unhelpful thought that led to their doing something wrong: for example, ‘I want to play with the ball that boy is playing with. If I hurt him, he will stop playing with the ball, then I can use it to start my own game of football.’ The adult then continues by helping the child to recognise how their antisocial behaviour has a direct impact on other people's thoughts and willingness to accommodate the child's wishes and needs. For example, in this instance, the adult helped the child to understand that his behaviour made the teacher who witnessed him hurting the boy think he wasn't able to play football safely, as a consequence of which she took the ball away from him. From this point, the child was able to shift his thinking and, therefore, his behaviour to a ‘Smarter’ position, by thinking, ‘I want to be able to play football at play time. My teacher needs to see that I can keep my body and voice smart, so then she will think that I will be able to play football safely’. The child's Smart Thought–Behaviour Chain which was recorded during the session was then shared with other staff members, so they could give him prompts and feedback at playtime, using Smart Language to encourage him to use and extend his Smart Thinking, and Smart Behaviour. Staff were trained to use LGS Smart Language phrases, e.g. ‘As soon as I can see that your body is SMART, I will be able to let you play football …’, ‘If you finish your writing, I will be pleased with you and this will make me want to let you play football at playtime’.

Further information on LGS is available from Dr Laura Warren (e-mail: laurawarren01@aol.co.uk)

## References

[R1] Boxall M (2002). Nurture groups in school: Principle and practice.

[R2] Butterworth B., Varma S., Laurillard D. (2011). Dyscalculia: From brain to education. Science.

[R3] Carroll J.M., Maughan B., Goodman R., Meltzer H. (2005). Literacy difficulties and psychiatric disorders: Evidence for comorbidity. Journal of Child Psychol Psychiatry.

[R4] Creswell J.W., Plano Clark V.L. (2007). Designing and conducting mixed methods research.

[R5] Dadds M.R., Jambrak J., Pasalich D., Hawes D.J., Brennan J. (2011). Impaired attention to the eyes of attachment figures and the developmental origins of psychopathy. Journal of Child Psychology and Psychiatry.

[R6] Finger E.C., Marsh A.A., Mitchell D.G., Reid M.E., Sims C., Budhani S., Kosson D.S., Chen G., Towbin K.E., Leibenluft E., Pine D.S., Blair R.J.R. (2008). Abnormal ventromedial prefrontal cortex function in children with psychopathic traits during reversal learning. Archives of General Psychiatry.

[R7] Finger E.C., Marsh A.A., Blair K.S., Reid M.E., Sims C., Ng P., Pine D.S., Blair R.J.R. (2011). Disrupted reinforcement signaling in the orbitofrontal cortex and caudate in youths with conduct disorder or oppositional defiant disorder and a high level of psychopathic traits. American Journal of Psychiatry.

[R8] Geake J (2008). Neuromythologies in education. Educational Research.

[R9] Gioia G.A., Isquith P.K., Guy S.C., Kenworthy L. (2000). Behavior rating inventory of executive function: Professional manual.

[R10] Goswami U (2006). Neuroscience and education: From research to practice?. Nature Reviews Neuroscience.

[R11] Goswami U., Szucs D. (2011). Educational neuroscience: Developmental mechanisms: Towards a conceptual framework. NeuroImage.

[R12] Greene R.W., Ablon J.S. (2006). Treating explosive kids: The collaborative problem-solving approach.

[R13] Haas S.M., Waschbusch D.A., Pelham W.E., King S., Andrade B.F., Carrey N.J. (2011). Treatment response in CP/ADHD children with callous/unemotional traits. Journal of Abnormal Child Psychology.

[R14] Hawes D.J., Dadds M.R. (2005). The treatment of conduct problems in children with callous-unemotional traits. Journal of Consulting and Clinical Psychology.

[R15] Jones A.P., Laurens K.R., Herba C.M., Barker G.J., Viding E. (2009). Amygdala hypoactivity to fearful faces in boys with conduct problems and callous-unemotional traits. American Journal of Psychiatry.

[R16] Kazdin A.E., Kazdin A.E., Weisz J.R. (2003). Problem-solving skills training and parent management training for conduct disorder. Evidence-based psychotherapies for children and adolescents.

[R17] Kennedy J.H., Kennedy C.E. (2004). Attachment theory: Implications for school psychology. Psychology in the Schools.

[R18] Kimonis E.R., Frick P.J., Skeem J., Marsee M.A., Cruise K., Munoz L.C., Aucoin K.J., Morris A.S. (2008). Assessing callous-unemotional traits in adolescent offenders: Validation of the Inventory of Callous-Unemotional Traits. Journal of the International Association of Psychiatry and Law.

[R19] Krätzig G., Arbuthnott K. (2006). Perceptual learning style and learning proficiency: A test of the hypothesis. Journal of Educational Psychology.

[R20] Law J., Plunkett C. (2009). The interaction between behaviour and speech and language difficulties: does intervention for one affect outcomes in the other? Technical report. Research evidence in education library.

[R21] Mattison R.E., Rutherford R.B., Quinn M.M., Mathur S.R. (2004). Psychiatric and psychological assessment of emotional and behavioral disorders during school mental health consultation. Handbook of research in emotional and behavioral disorders.

[R22] McCabe D.P., Castel A.D. (2008). Seeing is believing: The effect of brain images on judgments of scientific reasoning. Cognition.

[R23] McCloskey G., Perkins L.A., Van Divner B. (2009). Assessment and intervention for executive function difficulties.

[R24] McCrae S.M., Mueller J.H., Parrila R.K. (1999). Quantitative analyses of schooling effects on executive function in young children. Child Neuropsychology.

[R25] Muhr T (2004). User's manual for ATLAS.ti 5.0.

[R26] Pickering S.J., Howard-Jones P. (2007). Educators’ views on the role of neuroscience in education: Findings from a study of UK and international perspectives. Mind, Brain, and Education.

[R27] Reynolds C.R., Kamphaus R.W. (2004). Behavior assessment system for children.

[R28] Roose A., Bijttebier P., Decoene S., Claes L., Frick P.J. (2010). Assessing the affective features of psychopathy in adolescence: A further validation of the inventory of callous and unemotional traits. Assessment.

[R29] Simos P.G., Fletcher J.M., Sarkari S., Billingsley R.L., Denton C., Papanicolaou A.C. (2007). Altering the brain circuits for reading through intervention: A magnetic source imaging study. Neuropsychology.

[R30] Sterzer P., Stadler C. (2009). Neuromaging of aggressive and violent behaviour in children and adolescents. Frontiers in Behavioral Neuroscience.

[R31] Turkeltaub P., Gareau L., Flowers D.L., Zeffiro T.A., Eden G.F. (2003). Development of neural mechanisms for reading. Nature Neuroscience.

[R32] Varma S., McCandliss B.D., Schwartz D.L. (2008). Scientific and pragmatic challenges for bridging education and neuroscience. Educational Researcher.

[R33] Vaughn M.G., DeLisi M., Beaver K.M., Wexler J., Barth A., Fletcher J. (2011). Juvenile psychopathic personality traits are associated with poor reading achievement. Psychiatric Quarterly.

[R34] Vaughn S., Schumm J.S., Sinagub J. (1996). Focus group interviews in education and psychology.

[R35] Viding E., Jones A.P. (2008). Cognition to genes via the brain in the study of conduct disorder. Quarterly Journal of Experimental Psychology.

[R36] Viding E., Blair R.J.R., Moffitt T.E., Plomin R. (2005). Evidence for substantial genetic risk for psychopathy in 7-year-olds. Journal of Child Psychology and Psychiatry.

[R37] Viding E., McCrory E.J., Blakemore S.J., Frederickson N. (2011). Behavioural problems and bullying at school: Can cognitive neuroscience shed new light on an old problem?. Trends in Cognitive Science.

[R38] Wengraf T (2001). Qualitative research interviewing: Biographic narratives and semi-structured methods.

